# Assessment of Microbiological Quality and Mycotoxin in Dried Chili by Morphological Identification, Molecular Detection, and Chromatography Analysis

**DOI:** 10.3390/ijerph17061847

**Published:** 2020-03-12

**Authors:** Rachma Wikandari, Inggrid Chrisanti Mayningsih, Maura Dania Permata Sari, Fiametta Ayu Purwandari, Widiastuti Setyaningsih, Endang Sutriswati Rahayu, Mohammad J. Taherzadeh

**Affiliations:** 1 Department of Food and Agricultural Product Technology, Gadjah Mada University, Yogyakarta 55281, Indonesia; Rachma_wikandari@mail.ugm.ac.id (R.W.); inggridchrisanti@gmail.com (I.C.M.); Mauradaniaps@gmail.com (M.D.P.S.); fiametta@ugm.ac.id (F.A.P.); widiastuti.setyaningsih@ugm.ac.id (W.S.); endangsrahayu@ugm.ac.id (E.S.R.); 2 Swedish Centre for Resource Recovery, University of Borås, 50190 Borås, Sweden

**Keywords:** aflatoxin, ochratoxin A, dried chili, Indonesia, molecular, mycobiota

## Abstract

The growing interest in spicy foods leads to the global demand for spices, particularly dried chili. This study aimed to assay both aflatoxin (AFs) and ochratoxin A (OTA) contamination using an integrative method of morphological identification, molecular detection, and chromatography analysis on dried chili provided from traditional and modern markets in Indonesia. The results showed that total fungal infection ranged from 1-408 × 10^3^ CFU/g. Eighty percent of the chili obtained from both the traditional and the modern markets were infected by *Aspergillus* spp., in which 50% of the infections were identified as *A. parasiticus* and *A. flavus*. A complete set of targeted genes involved in AF production and OTA were detected in two isolates of *A. flavus* and one isolate of *A. carbonarius*, respectively. The levels of AFs B_1_, B_2_, and OTA in the contaminated dried chilies were in the range of 39.3–139.5 µg/kg, 2.6–33.3 µg/kg, and 23.7–84.6 µg/kg, respectively. In contrast, no AFs G_1_ and G_2_ were detected. This study showed that the fungal infection of Indonesian dried chili occurs both in the field and during storage; thus, it is suggested to implement good agricultural and handling processes.

## 1. Introduction

Spicy food has been gaining global attention, and this has promoted an increase of demand for spices in the market, notably chili. Chili is a fruit of the *Capsicum* plant belonging to the *Solanaceae* genus, which is originated from America. Chili is an essential ingredient which is added to main dishes or sauces worldwide. Besides its culinary application, there has been growing interest in utilizing chili for medical purposes such as in pain killers and anticancer agents. Chili is available in the market in the form of fresh and dried products. There are several types of dried chili, such as whole chili, in flakes, and in ground powder. China is the main supplier of dried whole and ground chili in the European market [1]. However, according to a report from the Rapid Alert System for Food and Feed (RASFF), approximately 36 % of the alerts in the last fifteen years for dried chili from China are due to mycotoxin contamination [2].

Mycotoxins are the secondary metabolites produced by fungi that have detrimental effects on human and animal health. Among the 400 known mycotoxins, aflatoxin (AFs) and ochratoxin (OTA) pose the primary concern to animal and human health. AFs are mainly produced by *Aspergillus flavus* and *Aspergillus parasiticus* and are the most potent natural carcinogenic found in nature. AFs pose several toxic effects in animals and humans, such as carcinogenic, mutagenic, teratogenic, and immunosuppressive activity [3]. The most common AFs present in food are B_1_, B_2_, G_1_, and G_2_. AFB_1_ has the highest genotoxic and carcinogenic effect, as well as being the most commonly found AF in agricultural products [4]. AFB_1_ and AFs have been classified as group I carcinogens (carcinogenic to humans) by the International Agency for Research on Cancer (IARC) [5]. OTA, which is produced by *Penicillium verrucosum*, *A. ochraceus*, and *A. niger* [6], is reported to cause chronic kidney disease and may be associated with the development of gallbladder cancer [7]. Similar to AFs, the toxic effects of OTA against various experimental animals include carcinogenic, teratogenic, immunotoxic, genotoxic, and possibly neurotoxic activity [8]. According to IARC, OTA has been classified as a group 2B carcinogen based on sufficient evidence for carcinogenicity in animal studies and inadequate evidence in humans [5]. 

Mycotoxin can be produced in a crop during pre and post-harvest. Several factors influence mycotoxin production during pre-harvest, including the type of soil and crop as well as the amount of *Aspergillus* contaminating the plant [9]. Besides this, the higher temperature, humidity, and heavy rainfall result in a higher risk of mycotoxin contamination [9]. Similarly, higher temperature and humidity during storage also increase the risk of AF production post-harvest [9]. Hence, crops of tropical countries are more prone to AF contamination due to their climate, with high temperatures and moisture, monsoons, unseasonal rains during harvest, and flash floods [10].

Several studies have reported mycotoxin contamination in chilis from Turkey, Pakistan, Malaysia, Bangladesh, Qatar, and Saudi Arabia [11,12,13,14,15,16]. However, most of these studies only assessed the mycotoxin level, and few studies estimated the fungal contamination or molecular tool for the detection of toxigenic fungi. Additionally, another report showed OTA contamination; the rest only focus on AFs [13]. To the best of our knowledge, a combination of morphological identification, molecular tools, and the chromatographic technique for the identification and quantification of both AFs and OTA in contaminated foods has never been reported. The integration of these approaches is particularly important to give a better insight into recognizing the potential risk for both AF and OTA contamination. 

In this study, therefore, we used integrative approaches consisting of morphological, molecular, and chromatography techniques to assay both AF and OTA contamination in dried chili marketed in Indonesia. Indonesia is a tropical country, with a climate which is susceptible to mold growth and mycotoxin production. Although Indonesia is the largest chili producer in Southeast Asia and the fourth largest in the world, contributing 5.8% of global production [17], mycotoxin contamination in Indonesian dried chili has not yet been reported. Our samples were taken from two different types of markets—i.e., traditional and modern markets—to study the effect of sanitation on the microbiological quality of the dried chili.

## 2. Materials and Methods 

### 2.1. Materials

The dried chilies used in this study were purchased randomly from both the traditional and the modern markets in the form of whole, ground, and powdered samples. The cultivars of chili samples provided from the traditional markets were green cayenne chili, white bird’s eye chili, red pepper, red bird’s eye chili, and red cayenne chili. Six samples from the traditional markets were in the form of dried chili, and two samples were in the form of powdered chili. The samples taken from the modern market included six powdered chilies and one dried chili from different brands. The samples were stored at 4 °C after purchase. 

### 2.2. Chemicals and Reagents 

All chemicals were of analytical reagent grade, except for methanol and acetonitrile, which were of high-performance liquid chromatography (HPLC) grade. All the chemicals were purchased from Merck (Darmstadt, Germany). All solutions were prepared using de-ionized water. AFs and OTA standards were purchased from Trilogy Analytical Laboratory (Washington, Missouri). Each standard of AFs contained 5 µg/mL of AFB1, AFB2, AFG1, AFG2, and the concentration of OTA was 1 µg/mL.

### 2.3. Fungal Occurrence in Chilis 

To investigate the fungal occurrence in the dried chili, 10 g of each sample was mixed with 90 mL of sterile NaCl 0.85%. Each mixture was shaken vigorously. The fungus culture method was based on Rahayu et al. [18]. The samples were diluted by a factor of 10, 100, and 1000. Subsequently, 0.1 mL of the mixture for each dilution was aseptically inoculated onto a Dichloran-18 Glycerol (DG-18) (Oxoid Ltd., Hants, UK) agar plate with 0.01% chloramphenicol (Merck, Darmstadt, Germany). The plates were then incubated at 25 °C for 5–7 days. After 2–3 days of incubation, the growing fungal colonies were counted, and at the end of incubation, the color of the colony was observed. The frequency of fungal occurrence was calculated as colony-forming units (CFU) per gram of samples. 

### 2.4. Isolation and Identification of Fungi from Dried Chili and Chili Powder

Each formed colony with a green and black color in DG-18 agar was suspected as *Aspergillus* and thus transferred into Malt Extract Agar (MEA) (Oxoid Ltd., Hants, UK) with 0.01% chloramphenicol (Merck, Darmstadt, Germany). The isolation of the molds was carried out based on the methods of Samson et al. [19]. For identification, each isolate was grown in MEA and Czapek Yeast Extract Agar (Merck, Darmstadt, Germany) medium with 0.01% chloramphenicol (Merck, Darmstadt, Germany). The isolates were identified based on their micro and macromorphology [18,20,21].

### 2.5. Molecular Identification of AF and OTA-Producing Isolates

Fungal DNA was extracted using an Invisorb^®^ Spin Plant Mini Kit (Stratec Molecular Gmbh, Berlin, Germany). The DNA was then amplified using PCR with the primers nor-1, aflR, and omtB to screen for the AF biosynthesis gene. To screen for the OTA biosynthesis gene, the polyketide gene was amplified using the primers AcPKS and AnPKS. All primers were ordered from Integrated DNA Technologies (IDT, Coralville, Iowa). The sequence of the primers used for amplification is shown in Table 1. The reaction of PCR amplification was performed in 25 µL of mix PCR (GoTaq^®^ Master Mixes, Promega, Madison, USA) mixed with 21 µL distilled water, 1 µL of each primer, and 1 µL of DNA template. The PCR program is presented in Table 2.

### 2.6. Mycotoxin Extraction from Dried Chili

The whole chili samples were first grounded. For AF extraction, ground chili (25 g) was blended with 125 mL of methanol/water (80/20 v/v) and 2.5 g NaCl for 30 min. The mixture was then incubated in a shaker for 1 h. Subsequently, 125 mL of distilled water was added to the mixture, and the mixture was filtrated through a filter paper (Whatman grade 4, Whatman International Ltd., Maidstone, UK). The filtrate (10 mL) was diluted with 20 mL of phosphate-buffered saline (PBS), and the pH was adjusted to 7.2 using 2 M NaOH. For purification, the diluted filtrate (10 mL) was then subjected through an immunoaffinity column (IAC) (Aflaprep PO7, R-Biopharm RhÔne Ltd., Glasgow, UK) at a flow rate of 2 mL per minute. To remove undesired compounds, 20 mL of PBS was passed through the IAC at 5 mL/min. To elute the AFs, 500 µL of methanol and water (80/20 v/v) was slowly passed through the column. The elute was collected in a tube, and this process was repeated until 1 mL of elute was obtained. The methanol was then evaporated using nitrogen until the dry sample was obtained. Subsequently, the sample was reconstituted in 20 μL of mobile phase. For OTA extraction, ground samples (12.5 g) were mixed with 100 mL of NaHCO_3_ 1% and incubated in a shaker for 1 h. The mixture was centrifuged at 3500 rpm for 10 min, and the mixture was filtrated through a filter paper (Whatman grade 4, Whatman International Ltd., Maidstone, UK). PBS (8 mL) was then added to the filtrate and homogenized. The pH was adjusted to 7.2 by 2 M NaOH, and the mixture was passed through an immune affinity column (Ocraprep, PO7, R-Biopharm RhÔne Ltd., Glasgow, UK). PBS (20 mL) with a pH of 7.2 was slowly passed through the IAC to remove undesired compounds. Subsequently, 1.5 mL of MeOH: glacial acetic acid (98:2) was added to the column and incubated for 5 min. The samples were dried with nitrogen to remove the methanol. The sample was then reconstituted in 400 µL of mobile phase and subjected to analysis by HPLC.

### 2.7. Mycotoxin Determination by HPLC 

The AF and OTA concentrations were analyzed by high-performance liquid chromatography (HPLC) (Shimadzu LC-20 AD, Kyoto, Japan) using a C18-analytical column (LiChrosphere 100 RP-C18e, Merck, Darmstadt, Germany) and fluorescence detector. An isocratic mobile phase for AF analysis was methanol/water (40:60 v/v) adjusted with 350 µL of 4 M nitric acid and 119 mg of HNO_3_ and 119 mg KBr per 1 L of mobile phase. The mobile phase for OTA analysis was acetonitrile/distilled water/glacial acetic acid (45:52.5:25 v/v). A sample (20 µL) was injected into the HPLC system with a flow rate of 1 mL/min. The fluorescence detector was set with excitation and emission wavelengths of the detector of 350 and 450 nm, respectively, for AF analysis. In contrast, for OTA analysis, they were set at 336 nm and 440 nm, respectively. The analytical column temperature was maintained at 40 °C for both AF and OTA analysis. The samples were injected into an HPLC column and then subjected to post-column derivatization (Kobra Cell, R-Biopharm Rhône Ltd., Glasgow, UK). The limits of detection (LODs) were 0.61 µg/kg for AFB_1_, 0.05 µg/kg for AFB_2_, 0.553 µg/kg for AFG_1_, 0.06 µg/kg for AFG_2_ and 0.53 µg/kg for OTA. The standard curve was linear at six concentrations between 12.5 and 400 ng/mL. The *R*^2^ values for AFs G2, G1, B2, and B1 were 0.996, 0.997, 0.996, and 0.996, respectively. 

### 2.8. Experimental Design and Data Analysis

A non-factorial experiment was conducted to evaluate the fungal contamination of dried chili purchased from traditional and modern markets. The single factor ANOVA was calculated to determine the effect of different markets on the contamination (*p* < 0.05).

## 3. Results

### 3.1. Microbiota of Dried and Powdered Chili

**Figure 1:**
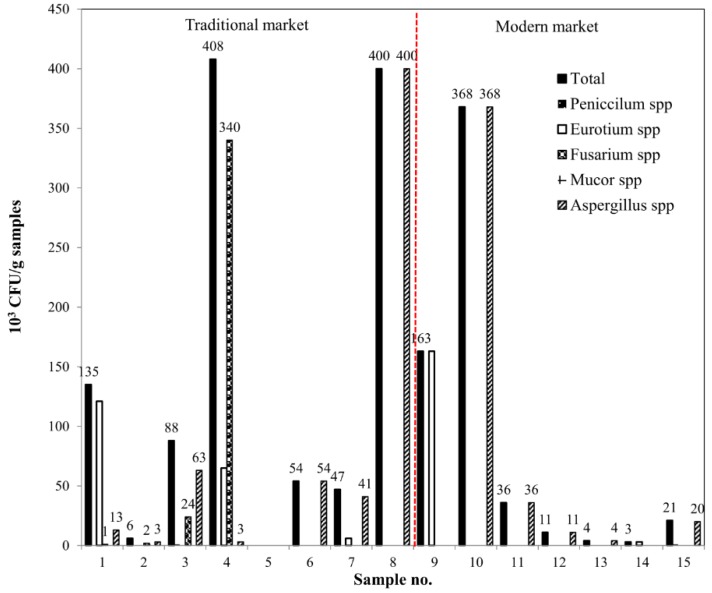
Microbiota of dried and powdered chili from the traditional and the modern market.

The microbiota of dried chili cultivated in DG-18 agar plates are presented in Figure 1. The fungal contamination levels of the chilies ranged from 1 to 408 × 10^3^ CFU/g. The level of fungal contamination in this study did not exceed the range reported in previous studies. In Saudi Arabia, total counts of fungi isolated from red pepper were 1.8–1.5 × 10^3^ CFU/g [25]. In Spain, fungal counts were between <0.1 × 10^3^ and 46 × 10^3^ CFU/g in *Capsicum* powder [26]. In Iran, mold and yeast contamination was between 24 × 10^3^ and 4600 × 10^3^ CFU/g in Iranian red pepper spice [27]. In Korea, fungal contamination in ground red pepper was 7.3 × 10^3^ CFU/g [28]. The contamination level for the traditional market on average (142 × 10^3^ CFU/g) was not significantly different from that purchased from the modern market (87 × 10^3^ CFU/g). In addition, the microbiota of chili purchased from the traditional market was more diverse than in the modern market. Dried chili purchased from the traditional market was contaminated with five genera (*Penicillium* spp., *Eurotium* spp., *Fusarium* spp., *Mucor* spp. and *Aspergillus* spp.); meanwhile, only three genera (*Penicillium* spp., *Eurotium* spp. and *Aspergillus* spp.) were detected in dried chili purchased in the modern market. The results of this work were in agreement with a previous study reporting that *Aspergillus*, *Penicillium*, and *Fusarium* are commonly isolated fungi from red pepper [25].

**Figure 2:**
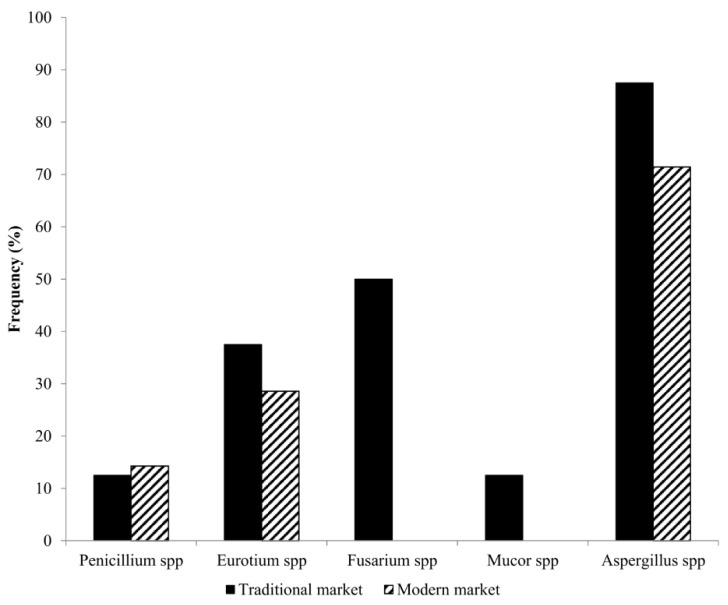
Frequency of fungal genera detected in dried and powdered chili from the traditional and the modern market.

The frequencies of contamination of each genus, excluding *Penicillium* spp., were higher in dried chili from the traditional market than the modern market (Figure 2). For instance, the percentages of contaminated dried chilies by *Eurotium* spp. and *Aspergillus* spp. were 37.5% and 87.5%, respectively, in the traditional market compared to 28.17% and 71.43% in the modern market, respectively The higher amount of microbiota diversity and occurrence frequency of the fungal contamination in the traditional market compared to the modern market could be explained by the lower sanitary and quality standards applied to dried chili in the traditional market. The result of the current study showed that both storage molds and field fungi were found in samples purchased from the traditional market, which indicates that the contamination of the products may occur both in the field and/or during storage. One explanation for this could be that the buildings of the traditional market are often semi-open-air, without a partition between each seller for different commodities, thus allowing street dust and cross-contamination to occur during the display of the products. In addition, sun-drying is the most common practice for drying the chili in the traditional market; thus, the risk of contamination from soil and air increases. In contrast, the contamination in chili purchased from the modern market might only occur during storage, as no *Fusarium* spp. was detected. 

### 3.2. Identification of Aspergillus Spp. Fungi on Dried Chili and Chili Powder

**Figure 3:**
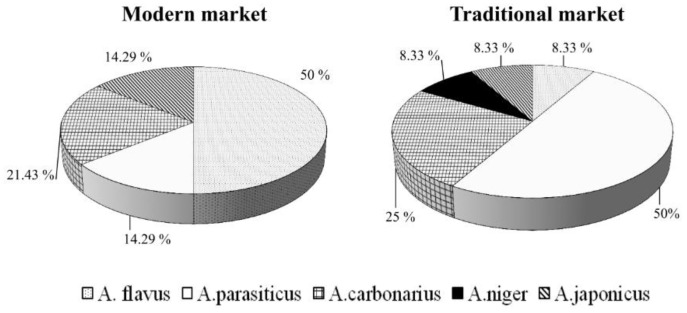
Frequency of *Aspergillus* spp. detected in dried and powdered chili from the traditional and the modern markets.

In general, 80% of the samples were contaminated by *Aspergillus* spp. The contaminations by these fungi were in the range of 3–400 × 10^3^ CFU/g in the traditional market and 4–368 × 10^3^ CFU/g in the modern market. The isolates identified as *Aspergillus* spp. cultivated in DG-18 agar were cultivated in MEA and CYA for further identification to the species level based on their micro and macromorphology. The result shows that the total number of isolates of *Aspergillus* spp. found in all samples was 26 (Table 3). The species of *Aspergillus* detected in the samples were *A. flavus*, *A. parasiticus*, *A. carbonarius*, *A. niger*, and *A. japonicus*. All of these species were detected in dried chili taken from the traditional market. However, *A. niger* was absent in the sample taken from the modern market. In general, the samples were dominated by *A. flavus* and *A. parasiticus*, followed by *A. carbonarius*, *A. japonicus*, and *A. niger*. Dried chili purchased from the traditional market was dominated by *A. parasiticus*, followed by *A. carbonarius*, and the rest were present in the same frequencies (Figure 3). Meanwhile, dried chili purchased from the modern market was dominated by *A. flavus*, followed by *A. carbonarius*, and others found in the same frequencies. This result is in accordance with a previous study [29] which reported that *A. flavus* and *A. niger* were the most dominant fungi in red chili imported from various countries. The species of *Aspergillus* detected in red pepper in Saudi Arabia were *A. flavus*, *A. niger*, *A. ochraceus* and *A.fumigatus* [25]. It has been reported that *Aspergillus* section *nigri* was dominant in the chili powder sample [26].

The high frequency (50%) of *A. parasiticus* and *A. flavus* both in the traditional and the modern markets indicated the risk of mycotoxin contamination in the sample as *A. flavus* and *A. parasiticus* are commonly known as AF producers. In addition, 70% of the chili samples were infected by two and three species. The high co-occurrence of *Aspergilli* in this has been suggested to increase the chances of mycotoxin contamination. The high co-occurrence of *Aspergilli* was also reported on chili samples from Taif City, Saudia Arabia [16], whereas the co-occurrence of *Aspergilli* in sample chili from Spain was 35.5% [30].

### 3.3. Molecular Detection of Mycotoxin Production Genes 

In order to investigate the potential of AF and OTA production, two isolates of *A. flavus* and one isolate of each stain of *A. parasiticus*, *A. japonicus*, *A. carbonarius*, and *A. niger* isolated from the chili samples were examined by PCR. The PCR was used for the detection of aflatoxigenic *Aspergilli* based on the intermediated enzymes, including the norsolorinic acid reductase-encoding gene *nor-1*, the sterigmatocystin O-methyltransferase encoding gene *omt1* and the regulatory gene *aflR* [31]. *Nor-1* gene plays a role at the beginning of AF biosynthesis by converting norsolorinic acid into averantin [32]. The *aflR* gene plays an essential role in the AF biosynthetic pathway by regulating the activity of other structural genes such as *omt-A, ver-1* and *nor-1* [33,34]. The *omtB* gene plays a role at the end of AF biosynthesis by producing O-methyltransferase, which converts dimethylsterigmatocystin into sterigmatocystin, which will be further transformed into AFB_1_. 

Table 4 showed that the *nor-1* gene was detected in *A. niger* isolate, 2 *A. flavus* isolates, and *A. parasiticus*. A targeted gene of *aflR* was detected on all isolates, whereas DNA fragments corresponded to *omtB* gene were only detected on two isolates of *A. flavus* and isolate of *A. japonicus*. A complete set of the genes was only detected on two isolates of *A. flavus*. To detect the potential OTA production, the primers AnPKS and AcPKS, which are specific to amplifying the DNA of the isolates of *A. niger* and *A. carbonarius*, respectively, were used. The results showed that the targeted gene was present in *A. carbonarius* but absent in *A. niger*.

It has been suggested that AF biosynthesis is a complex pathway involving the pattern of positive-and negative-acting transcriptional regulatory factors affected by environmental and nutritional parameters [35,36]. Thus, AF production does not only depend on the presence of the gene involved in AFs production but also the environmental condition that favors the production of AFs. Thus, to confirm the occurrence of AFs in the chili sample, the AF concentration on the sample should be determined.

### 3.4. AF and OTA Concentration on Dried Chili and Chili Powder 

To investigate the AF and OTA contamination on dried chili, six samples were randomly selected and examined using HPLC. The results showed that two samples were not contaminated by AFs and OTA, one sample was only contaminated with AFs or OTA and two samples were contaminated by both AFs and OTA. The levels of the AFs B_1_ and B_2_ in the contaminated dried chilies were in the range of 39.3–139.5 and 2.6–33.3 µg/kg, whereas no AFs G_1_ and G_2_ were detected (Table 5). The level of OTA in contaminated chilies was in the range of 23.7–84.6 µg/kg (Table 5). The AFs in sample 2 might be produced by *A. parasiticus* (Table 3). Sample 3 was contaminated by OTA, which might be produced by *A. carbonarius* (Table 3). No OTA was produced in sample 2, and this corresponds to the absence of the *AnPKS* gene. Although *A. parasiticus* and *A. carbonarius* (Table 3) were found in sample 6, no AF or OTA was produced. Similarly, no AF and OTA was detected in sample 8 containing *A. parasiticus* and *A. japonicus* (Table 3). This might be explained by the environmental and nutrition conditions that do not favor the production of AFs and OTA in this sample. Sample 12 was contaminated by OTA, which might be produced by *A. carbonarius* as it contained the *AcPKS* gene.

Interestingly, AFs were also found in sample 12, which was not expected. This might be produced from *Aspergillus flavi*, which might also contaminate the sample. A high amount of AF was found in sample 15, which was expected, as *A. flavus* showed the complete set of the genes, and sample 15 contained four instances of *A. flavus* and one of *A. parasiticus* (Table 3). Interestingly, OTA was also found in this sample. This indicates that the sample might also be contaminated by OTA-producing fungi. The co-occurrence of different mycotoxins in one product raises the chance of synergetic interaction, which might result in more adverse impacts on human health [37]. The results showed that some of the dried chilies were contaminated by AFs and OTA simultaneously. This might lead to a possibly higher risk for adverse health effects, as demonstrated by the fact that OTA can raise the mutagenicity of AFB_1_ once they have infected the same substrate. 

## 4. Discussion

### Is Indonesian Chili more Contaminated than in Other Countries?

Chili is the second largest commodity after black pepper, contributing to 16% of the world spice trade [12]. The global production of chili and pepper crops comes to around 32 million tons, which are cultivated on approximately 1.9 million ha of land [17]. Chilis have been reported to be one of the commodities with the highest AF contamination [38]. In this work, 15 samples of dried chili, including eight samples from a traditional market and seven of the most consumed trademarks available in the modern markets, were examined for their microbiota, the occurrence of potential mycotoxigenic fungi, the molecular detection of potential mycotoxin producing isolate and mycotoxins level in the samples. 

The levels of AF and OTA contamination in Indonesian dried chili obtained in this study were in the range of those reported from previous studies [12,13,14,15,16,39]. Contamination levels of AFs B_1_ on the whole and ground chilies from Pakistan were 0–96.3 µg/kg and 0–89.56 µg/kg, respectively [12]. Set and Erkmen [11] reported that 120 samples of ground red chili peppers sold in Gaziantep, Turkey were found to be contaminated with total AFs and AFs B_1_, in which the number of samples containing AFs B_1_ over the legal limit in Turkey was higher in unpacked (62.5%) than that of packed ground red chili (9.4%). Jalili and Jinap reported that 65% of chili purchased from modern markets and traditional markets in Malaysia were contaminated by AFs, with total AFs level in the range of 0.2–79.7 µg/kg [13]. They also reported that 81.25 % of the samples contained OTA in the range of 0.2-101.2 µg/kg. Roy et al. reported that AFs B_1_ was found in red chili taken from 3 cities in Bangladesh with AFs level > 20 µg/kg [14]. A combination of morphological, physiological, and molecular detection showed that chili imported to Qatar contained AFs B_1_, B_2_, and total AFs of 69.28, 1.73, and 71.1 µg/kg sample [15]. Gherbawy et al. analyzed the chili samples from Taif City, Saudia Arabia, and found that the chili powder was contaminated with AFs in the range of 20-170 µg/kg and 35-200 µg/kg for crushed and powdered chili, respectively [16]. Khan et al. reported that AFs contamination in whole, crushed, and powdered chili collected from all over Pakistan were 11.7, 27.8, and 31.2 μg/kg, respectively [39].

Many countries have established regulations for mycotoxin contamination in food due to its adverse effect on human health. Some countries have established a legal limit for both AFB_1_ and AFs for all foods. The maximum limits for AFB_1_ vary from 2 µg/kg in Tunisia to 20 µg/kg in Nigeria, and the limits for total AFs vary from 5 µg/kg in Cuba to 35 µg/kg in Malaysia. In the United States, the maximum level of AFB_1_ contamination permitted in human food is 20 µg/kg [40]. On the other hand, some countries have established a legal limit for certain products. For instance, Turkey and Europe set the legal limits of AFs in ground red chili pepper for total AFB_1_ and total AFS of 5 and 10 µg/kg, respectively [41,42]. In Korea, the maximum limit for ground red pepper of total AFB_1_, total AFs, and OTA were 10, 15, and 7 ng/g [28]. In Indonesia, the legal limit for AFB_1_, AFs, and OTA on chili has not yet established. However, a limit of 20 µg/kg for total AFs is allowed for the category “coconut, spices, and open drug medicines/herbs”. The result of the current study showed that the AF contaminations for some samples were higher than the legal limit. 

The result of this preliminary study showed that Indonesian chili has high a risk of AF contamination; thus, attempts should be made to prevent or lower the mycotoxin contamination, such as by using the method suggested by Set and Erkmen [11] including applying good agricultural practice and the prevention of soil contact during harvesting; transporting in clean containers; applying good personnel hygiene, as well as the removal of damaged peppers, hulls, leaves, and garbage during sorting; applying Good Manufacturing Processes during washing, seed removal and grinding; prevention of cross-contamination, drying to up to 10%–12% moisture content at 70–100 °C for 1–3 h in a dryer for the drying process; and applying vacuum packaging and storage under an air conditioner (below 65% RH and 20 °C). In addition, Duman suggested that storage in hermetic cubs lowers the risk of AF contamination [43].

## 5. Conclusions

In this study, the potential mycotoxin contamination of dried Indonesian chili was assayed using a combination of morphological, molecular detection, and chromatography techniques. The result of the morphological assays showed that *Aspergillus* spp. was the dominant fungi contaminating the chili, and the commonly found isolates were *A. flavus* and *A. parasiticus*, which are known to be AF producers. Molecular detection by PCR showed that several genes involved in AF and OTA biosynthesis were found in several isolates. In addition, some of the dried chilies were contaminated by AFs and OTA. The results of this study showed the potential risk of dried chili being contaminated by aflatoxigenic and ochratoxigenic fungi as well as AFs and OTA, and thus it is highly advisable to control the proper harvesting, drying, handling, storage, and transportation conditions to reduce this contamination.

## Figures and Tables

**Table 1:** Sequence of primers used for PCR analysis [22,23,24].

Genes	Forward Primer 5′-3′	Reverse Primer 5′-3′
*AflR*	CGCGCTCCCAGTCCCCTTGATT	CTTGTTCCCGATGACCA
*Nor-1*	ACCGCTCCGGCACTCTCGGCA	GTTGGCCGCCAGCTTCGACACAGC
*OmtB*	GCCTTGACATGGAAACCATC	CCAAGATGGCCTGCTCTTTA
*AcPKS*	GCAGCGGGAGTCAATGTAAT	GCGTCGTACAAAGCCTCTT
*AnPKS*	ACGGTAAACGTCCTGGATGA	CGTGCTGTTGAAGCCACTT

**Table 2:** Program of Polymerase Chain Reaction for amplification of genes involved in aflatoxin (AF) and ochratoxin A (OTA) production.

Genes	Cycle	Denaturation	Annealing	Extension	Final Extension
*aflR*	35	94 °C, 60 s	62 °C, 60 s	72 °C, 120 s	72 °C, 600 s
*nor-1*	30	95 °C, 60 s	65 °C, 120 s	72 °C, 240 s	72 °C, 600 s
*omtB*	35	94 °C, 60 s	55 °C, 60 s	72 °C, 60 s	72 °C, 600 s
*AnPKS*	35	95 °C, 10 s	52 °C, 10 s	72 °C, 15 s	72 °C, 300 s
*AcPKS*	35	95 °C, 10 s	62 °C, 10 s	72 °C, 15 s	72 °C, 300 s

**Table 3:** The occurrence of *Aspergillus* spp. detected in dried and powdered chili from the traditional and the modern market.

No.	Form, Type of Market	*A. flavus*	*A. parasiticus*	*A. carbonarius*	*A. niger*	*A. japonicus*
1	Powder, traditional	0	1	1	0	0
2	Powder, traditional	0	1	0	1	0
3	Whole, traditional	0	1	1	0	0
4	Whole, traditional	0	1	0	0	0
5	Whole, traditional	0	0	0	0	0
6	Whole, traditional	0	1	1	0	0
7	Whole, traditional	1	0	0	0	0
8	Whole, traditional	0	1	0	0	1
9	Powder, modern	0	0	0	0	0
10	Powder, modern	1	0	1	0	0
11	Powder, modern	1	1	0	0	1
12	Ground, modern	0	0	1	0	0
13	Powder, modern	1	0	1	0	1
14	Powder, modern	0	0	0	0	0
15	Powder, modern	4	1	0	0	0
Total		8	8	6	1	3
Frequency (%), n = 26		30.77	30.77	23.08	3.85	11.54

**Table 4:** Molecular characterization of *Aspergillus* strains isolated from dried and powdered chili purchased from the traditional and the modern markets.

No	Isolates	Species	aflR	OmtB	Nor-1	AnPKS	AcPKS
2	2B1	*Aspergillus niger*	+	-	+	-	-
11	11G1	*Aspergillus flavus*	+	+	+	n.d	n.d.
	11G2	*Aspergillus parasiticus*	+	-	+	n.d	n.d.
	11B1	*Aspergillus japonicus*	+	+	-	n.d	n.d.
12	12B1	*Aspergillus carbonarius*	+	-	-	-	+
15	15G5	*Aspergillus flavus*	+	+	+	n.d	n.d.

n.d. = not determined.

**Table 5:** AF concentration on dried chili and chili powder.

Sample	AFs (µg/kg)			OTA (µg/kg)
	B_1_	B_2_	G_1_ + G_2_	
2	139.5 ± 0.7	12.8 ± 0.5	n.d. ^*^	n.d.
3	n.d.	n.d.	n.d.	26.3 ± 0.7
6	n.d.	n.d.	n.d.	n.d.
8	n.d.	n.d.	n.d.	n.d.
12	39.3 ± 0.7	2.6 ± 0.5	n.d.	23.7 ± 0.7
15	100.1 ± 0.7	33.3 ± 0.5	n.d.	84.6 ± 0.7

* n.d. = not detected.
